# Inflammatory suppressive effect of prostate cancer cells with prolonged exposure to transforming growth factor β on macrophage-differentiated cells via downregulation of prostaglandin E_2_

**DOI:** 10.3892/ol.2014.2402

**Published:** 2014-08-01

**Authors:** AKINOBU HAYASHI, YOSHIFUMI S. HIROKAWA, MICHIKO KAGAYA, MASAYA FUJIWARA, MISAO YONEDA, KAZUKI KANAYAMA, KATSUNORI UCHIDA, KENICHIRO ISHII, TAIZO SHIRAISHI

**Affiliations:** 1 Department of Oncologic Pathology, Institute of Molecular and Experimental Medicine, Mie University Graduate School of Medicine, Tsu, Mie 514-8507, Japan; 2 Department of Clinical Nutrition, Faculty of Health Science, Suzuka University of Medical Science, Suzuka, Mie 510-0226, Japan

**Keywords:** prostate cancer, transforming growth factor β1, THP-1 macrophage, microenvironment

## Abstract

Transforming growth factor β1 (TGFβ1) regulates a variety of cellular functions, including cell growth, apoptosis and differentiation. The aim of the current study was to investigate the alterations of phenotypic events in the long-term exposure of prostate cancer (PCa) cells to TGFβ1 and its effect on macrophage-differentiated cells. The PCa cell line, PC-3, and the subclone, M1, were exposed to TGFβ1 for short- or long-term periods. TGFβ1 signaling was assessed by Smad3 phosphorylation, and non-canonical signaling was analyzed by quantitative polymerase chain reaction-based regulatory gene expression profiles. TGFβ1-exposed PCa cells were also co-cultured with phorbol 12-myristate 13-acetate (PMA)-treated THP-1 macrophages as a model of the tumor microenvironment. The phosphorylation of Smad3 in the PCa cells with long-term exposure was lower than that in the PCa cells with short-term exposure. Interleukin-6 mRNA expression in the PMA-treated THP-1 macrophages was significantly downregulated by co-culture with the PCa cells with long-term exposure. Cyclooxygenase-2 expression in the long-term TGFβ1-exposed PCa cells was lower than that in the control PCa cells, and the production of prostaglandin E_2_ (PGE_2_) in the long-term TGFβ1-exposed PCa cells was also significantly lower. The results of the current study demonstrated that the long-term TGFβ1 exposure of PCa cells induces phenotypic changes, including the downregulation of PGE_2_ production. This indicates that prolonged TGFβ-exposed PCa cells may change the cytokine production of macrophages in the tumor microenvironment.

## Introduction

Cancer and the surrounding stromal cells compose the tumor microenvironment that provides opportunities for reciprocal interactions between cancer, fibroblasts, inflammatory cells and microcapillary vessels. Cytokines, including chemokines, growth factors, angiogenic factors and prostaglandins, participate in these interactions. Among these cytokines, transforming growth factor β1 (TGFβ1) is a multifunctional growth factor produced by cancer cells, macrophages and fibroblasts, which exerts wide-ranging activities that affect cancer inhibition and promotion, epithelial mesenchymal transition (EMT), angiogenesis and the suppression of regulatory T cell function (1).

In human prostate cancer (PCa) tissues, TGFβ1 expression is increased compared with normal or benign prostate tissues, and is increased in PCa with lymph node metastasis compared with cancer without lymph node involvement (2). TGFβ1 suppresses PCa cell growth in a dose-dependent manner, but this inhibitory effect is lost at later stages (3,4). Collectively, TGFβ1 is considered to be a tumor promoter in PCa tissues.

Cancer and inflammation has long been studied in close connection with carcinogenesis and cancer development. Cyclooxygenase-2 (COX-2) is the major enzyme that converts arachidonic acid into prostanoids, which are involved in a number of pathological events, including inflammation and cancer progression (5). However, the mechanistic role of COX-2 in prostate carcinogenesis remains controversial. One study has shown that benign prostatic disease expresses higher COX-2 than PCa (6), while another study reported COX-2 overexpression in PCa (7).

Multiple inflammatory cells and mediators are involved in cancer-related inflammation and compose elements of the tumor microenvironment (8). Tumor-associated macrophages (TAM), which are derived from monocytes, infiltrate tumor tissue, promote the invasive capacity of cancer cells and in turn, metastasis, which is correlated with a poor prognosis in patients with prostate and breast cancer (9–12). The mechanism by which TAM promotes cancer promotion is considered to involve the production of angiogenic growth factors, proteases and cytokines, including TGFβ (13). The reciprocal interactions between macrophages and the various phenotypes of PCa in the tumor microenvironment may be diverse. To investigate this issue, the current study examined the tumor microenvironment model of PCa, and TGFβ and THP-1 macrophages, where the PCa cells were exposed to TGFβ for a long period of time. In addition, the cytokine mRNA from THP-1 macrophages and the regulatory factors from PCa were analyzed.

## Materials and methods

### Cell culture and reagents

The human PCa cell line, PC-3, and the subclone, M1 (14), were cultured in RPMI 1640 (R8758; Sigma-Aldrich, St. Louis, MO, USA) with 10% fetal bovine serum (FBS; Gibco-BRL, Carlsbad, CA, USA), and TGFβ1 (555–83601; Wako Pure Chemical Industries, Ltd., Osaka, Japan) was added to the culture medium at a final concentration of 10 ng/ml. The cell lines were cultured and passaged every seven days. TGFβ1 or vehicle was added to the culture medium simultaneously on each passage and kept in the medium until the next passage. The passage was repeated 10 times. TGFβ1 or vehicle long-term exposure for the PC3 and M1 cells was designated as TbL-PC3 or TbL-M1, and CoL-PC3 or CoL-M1, respectively. The cell culture of the short-term exposure was represented by overnight incubation of TGFβ1 or vehicle, and TGFβ1 was removed from the culture medium in subsequent experiments. Short-term exposure for the PC3 and M1 cells was designated as TbS-PC3 or TbS-M1, and CoS-PC3 or CoS-M1, respectively. The human acute monocytic leukemia-derived THP-1 cell line was maintained in RPMI 1640 medium supplemented with 10% FBS. The antibodies for western blot analysis and their dilutions were as follows: Rabbit polyclonal anti-human anti-Smad3 (ab28379; 1:1,000), rabbit monoclonal anti-human anti-phospho-Smad3 (S423+S425; ab52903, 1:1,000) and rabbit polyclonal anti-rat anti-COX-2 (ab15191; 1:1,000) (All Abcam, Cambridge, UK). The p3TP-Lux plasmid was kindly provided by Dr Joan Massague (15). The luciferase assay was performed as follows: The PC-3 cells were transfected with expression and reporter plasmids together with Lipofectamine (11668019; Invitrogen Life Technologies, Carlsbad, CA, USA) and harvested 24 h later. The firefly luciferase activity was counted using a Dual-Luciferase Reporter Assay System (E1910; Promega Corporation, Madison, WI, USA). Renilla luciferase activity was also estimated by cotransfection of the pRL-TK vector (E2241; Promega Corporation) as an internal control.

### Quantitative polymerase chain reaction (qPCR)

Total RNA was isolated from THP-1 macrophages using an RNA extraction kit (74104; Qiagen, Hilden, Germany). First-strand cDNA synthesis was performed using the Transcriptor High Fidelity cDNA Synthesis kit (05081963001; Roche Diagnostics GmbH, Mannheim, Germany). qPCR was performed with the QuantiTect SYBR Green PCR kit (204145; Qiagen) according to the manufacturer’s instructions. The results were analyzed by Rotor-GeneQ software (9020353; Qiagen) and normalized against GAPDH mRNA levels. The mRNA expression of 84 genes for human signal transduction molecules was analyzed by the RT^2^Profiler PCR Array (PAHS-014A; Qiagen), with the cDNA synthesis and SYBR Green PCR performed as aforementioned.

### Co-culture assay of the PCa cell line and THP-1 macrophages

The THP-1 cells were cultured at 5×10^5^ cells/well in 24-well plates and differentiated to THP-1 macrophages with 100 nm phorbol 12-myristate 13-acetate (PMA; P1585; Sigma-Aldrich) for two days. Following PMA removal from the culture media, the treated cells were maintained in RPMI 1640 with 10% FBS for an additional two days. The PC-3 cell line (without TGFβ1) was loaded in a cell culture insert (1.0-μM pore size; 353104; BD Falcon™ Cell Culture Inserts; BD Biosciences, Franklin Lakes, NJ, USA) at 1×10^4^ cells/300 μl medium and the inserts were placed in each well of a THP-1 macrophage culture plate. After two days of co-culture, total RNA was extracted from the THP-1 macrophages and cDNA was synthesized as aforementioned.

### qPCR primers

The primer sequences used were as follows: interleukin (IL)-6 forward, 5′-TCAGAACGAATTGACAAACA-3′ and reverse, 5′-TTGAATCCAGATTGGAAGC-3′; TNF-α forward, 5′-GACAAGCCTGTAGCCCATGT-3′ and reverse, 5′-TCTCAGCTCCACGCCATT-3′; and IL-10 forward, 5′-GCTGGAGGACTTTAAGGGTTACCT-3′ and reverse, 5′-CTTGATGTCTGGGTCTTGGTTCT-3′.

### Prostaglandin E_2_ (PGE_2_) production and enzyme immunoassay

All the cells were cultured at 5×10^5^ cells/well in triplicates of 24-well plates. The culture medium of the cells was changed to RPMI 1640 without FBS, but containing 10 μM of arachidonic acid (A3555; Sigma-Aldrich). Following 2 h of incubation, the media were collected from each well and PGE_2_ production was determined by the DetectX Prostaglandin E_2_ Enzyme Immunoassay kit (K018-H1; Arbor Assays, Ann Arbor, MI, USA).

## Results

### Smad3 phosphorylation status of PCa cells in response to short- and long-term TGFβ1 stimulation

To determine whether long-term TGFβ1 exposure can modify PCa cell signaling events, the PCa cell line, PC-3, and subclone, M1 (14), were used. Since IL-8 expression, which is regulated by TGFβ, is slightly different in PC-3 and M1 cells, we hypothesized that these cell lines may respond differently to TGFβ.

A growth inhibitory effect was observed when the PC-3 cells were exposed to TGFβ1 (4); PC-3 cells express TGFβ1 target genes (16), therefore, alterations in signaling caused by TGFβ1 stimuli should be observable.

When the PC-3 cells were incubated with TGFβ1, Smad2 C-terminal phosphorylation (Ser465/467) was induced, but was not robust (data not shown). In the majority of the commercially available antibodies, the C-terminal phosphorylated form of Smad2 and Smad3 is not distinguishable. The anti-phospho-Smad3 antibody, described in the Materials and methods section, does not cross-react with phospho-Smad2. Therefore, TGFβ1 signaling was evaluated using the phosphorylated status of Smad3.

**Figure 1:**
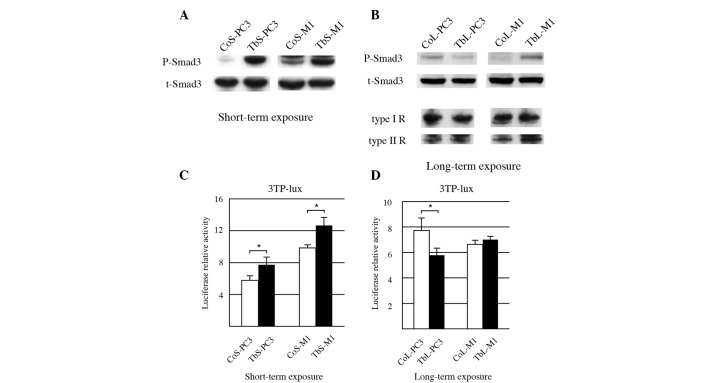
Smad3 phosphorylation status following short- and long-term TGFβ1 exposure and TGFβ1 target gene promoter activity. (A) Western blot analysis of P-Smad3 in short-term TGFβ1- or vehicle-exposed PC3 and M1 cell lines. Cell line designation of TGFβ1 or vehicle short-term exposure is described in the Materials and methods section. (B, upper) Western blot analysis of P-Smad3 in long-term TGFβ1- or vehicle-exposed cells. (B, lower) Protein expression with TGFβ types IR and IIR following TGFβ1 long-term exposure. (C and D) Smad3 target gene promoter activity was measured using the 3TP-lux luciferase reporter plasmid. Data are presented as the mean ± standard deviation of triplicate assays and represent two independent experiments. Student’s t-test was used to calculate statistical significances. ^*^P<0.05. TGFβ1, transforming growth factor β1; P-Smad3, phosphorylated Smad3; t-Smad3, total Smad3; type IR, type I receptor; type IIR, type II receptor.

As predicted, robust Smad3 phosphorylation was observed in the PC-3 and M1 cell lines tested following short-term TGFβ1 exposure (Fig. 1A). To further confirm Smad3 activity, a luciferase assay was conducted using the promoter for PAI-1, a Smad3 target gene. As shown in Fig. 1C, when the PC-3 and M1 cells were exposed to TGFβ1 in the short-term, the PAI-1 promoter activity was unregulated.

Following long-term exposure to TGFβ1, Smad3 phosphorylation in the TbL-PC3 and TbL-M1 cells was highly diminished compared with that in the vehicle-exposed cells (CoL-PC3 and CoL-M1), while TGFβ receptor expression was compatible between vehicle and TGFβ1 exposure (Fig. 1B). In contrast to the short-term exposure, PAI-1 promoter activity of the PC3 cells with long-term exposure was diminished compared with the cells exposed to the control treatment (Fig. 1D). PAI-1 promoter activity of the M1 cells with long-term exposure was at a similar level to that of the cells exposed to the control treatment (Fig. 1D). These results indicated that Smad signaling is attenuated in the PC-3 and M1 PCa cell lines exposed to long-term TGFβ1 treatment.

### Long-term TGFβ1 exposure of PCa cell suppresses cytokine production by THP-1 differentiated macrophages

To mimic the tumor microenvironment, the reciprocal interactions between TGFβ1-exposed PCa cells and inflammatory cells, in this case, macrophages, was examined. The activated macrophages were characterized with respect to the cytokines and receptors they produced and were designated as polarized macrophages (17). Since primary tissue macrophages are not easily obtainable, the human monocytic leukemia THP-1 cell line has been utilized in a number of studies (18–20). PMA treatment of THP-1 cells induces their differentiation into macrophage-like cells (THP-1 macrophages) that mimic the characteristics of monocyte-derived macrophages (21). As described in the Materials and methods section, cytokine production from THP-1 macrophages following reciprocal interactions with PCa cells was assessed by a chamber assay, where THP-1 macrophages and PCa cells were separated and could not make direct contact. When the THP-1 macrophages were co-cultured with the PC-3 cell line without any treatment, all cytokine production was increased, as previously described (22) (data not shown).

**Figure 2:**
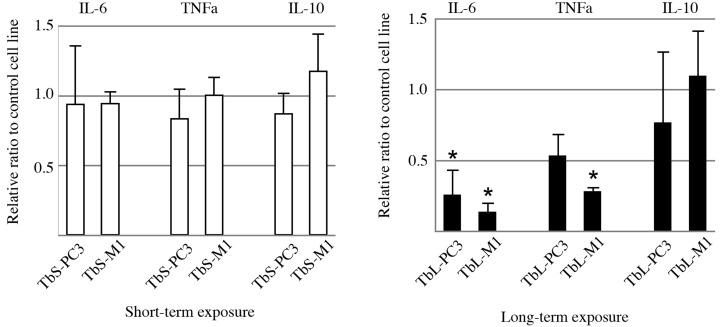
Cytokine production from THP-1 macrophages co-cultured with short- and long-term TGFβ1 exposure. Transcription levels of each cytokine from THP-1 macrophages with short- and long-term TGFβ1 exposure were measured by quantitative polymerase chain reaction. Relative ratios are exhibited as fold-changes compared with vehicle exposure. Data are presented as the mean ±standard deviation of triplicate assays and represent three independent experiments. Student’s t-test was used to calculate statistical significances. ^*^P<0.05 vs. vehicle exposure. TGFβ, transforming growth factor β.

IL-6 expression from the THP-1 macrophages was significantly decreased upon co-culturing with the PC-3 and M1 cells exposed to TGFβ1 for a long-term period (Fig. 2). TNF-α expression from the THP-1 macrophages was also markedly suppressed in the TbL-PC3 and TbL-M1 cells compared with the control cells. IL-10 expression was not altered significantly. However, IL-6 expression was increased, rather than decreased, by the addition of TGFβ1 to the THP-1 macrophage culture (data not shown).

Since IL-6 is a key regulator in PCa progression (23,24), the mechanisms of IL-6 downregulation in the THP-1 macrophages were investigated. Several growth factors, cytokines and prostanoids, such as hepatocyte growth factor (HGF), IL-1β, IL-4 and PGE_2_, have been found to regulate IL-6 production from macrophages or peripheral blood monocytes (25–27). Therefore, we hypothesized that TGFβ1 suppresses or stimulates pleiotropic factor secretion from PCa cells and consequently downregulates IL-6 production by THP-1 macrophages. Several of these potential factors were assessed at an mRNA level by qPCR (data not shown), which showed that HGF mRNA was unchanged in the CoL-PC3, TbL-PC3, CoL-M1 and TbL-M1 cells regardless of TGFβ1 exposure (data not shown). Although HGF was reported to downregulate IL-6 production from monocytic cell lines (25), this appears to have less relevance for IL-6 reduction in THP-1 macrophages.

**Figure 3:**
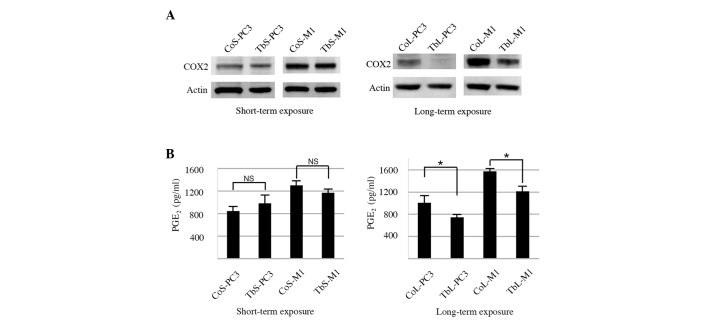
COX2 expression and PGE_2_ production by prostate cancer cells following short- and long-term exposure to transforming growth factor β1. (A) Cell lysates from each cell line were analyzed by western blot analysis using COX-2 antibody. (B) Each cell was treated with 10 μM arachidonic acid for 2 h, and PGE_2_ in medium was measured by an enzyme immunoassay. PGE_2_ levels are expressed as equal numbers of each cell type. Data are presented as the mean ± standard deviation of triplicate assays and represent two independent experiments. Student’s t-test was used to calculate statistical significances. ^*^P<0.05 vs. vehicle exposure (CoL-PC3 and CoL-M1). COX-2, cyclooxygenase-2; PGE_2_, prostaglandin E_2_.

Next, the possibility of non-canonical signal activity was explored in the cell lines with long-term TGFβ1 exposure through use of qPCR-based array analysis, which profiled the expression of key genes that are representative of various signal transduction pathways. Overall, the PCR array analysis showed that the TGFβ-related genes exhibited no marked changes in gene expression, with all changes observed being less than two-fold (Table I). Meanwhile, for the genes involved in the phospholipase C pathways, the expression of the COX-2 gene was downregulated in response to long-term TGFβ1 exposure. COX-2 protein expression was reduced by long-term TGFβ1 exposure in the TbL-PC3 and TbL-M1 cells (Fig. 3A).

PGE_2_ is known to induce IL-6 production from macrophages and is regulated by COX-2 activity (27), one of the rate-limiting enzymes for prostanoid biosynthesis (5).

The ability of the cells to produce PGE_2_ was also examined in the present study. The PGE_2_ level was not significantly different following short-term TGFβ1 exposure. However, following long-term TGFβ1 exposure, PGE_2_ production in the TbL-PC3 and TbL-M1 cells was reduced compared with that in the control cells (Fig. 3B).

Thus, these results indicated that COX-2 attenuation may be responsible for the reduction in PGE_2_ caused by long-term TGFβ1 exposure in TbL-PC3 and TbL-M1 cells, which consequently reduces IL-6 production in THP-1 macrophages.

## Discussion

In the current study, Smad signaling was shown to be diminished in the PCa cells following long-term TGFβ1 exposure. Cytokine production from THP-1 macrophages, particularly IL-6, was downregulated upon co-culture with PCa cells, producing lower levels of COX-2 and PGE_2_ by long-term TGFβ1 exposure.

The dynamic function of TGFβ1 allows it to be involved in a variety of intracellular signal transduction pathways (28). Several lines of evidence support the fact that a number of TGFβ1 signal transductions are independent from Smad canonical activation (29). The underlying mechanism for the reductions in phospho-Smad3 expression following long-term TGFβ1 exposure has not yet been fully elucidated. The turnover of phospho-Smad is mediated by the specific phosphatases PPM1A, PDP and SCP1, 2 and 3, or proteasomal degradation with the ubiquitin E3 ligase, NEDD4L (30–33). Neither the PPM1A transcript nor the NEDD4L protein expression were altered following exposure of the PCa cells to TGFβ1 or vehicle (data not shown). Other unidentified phosphatases or ubiquitin ligases may therefore be involved in the suppression of phospho-Smad3.

In the tumor microenvironment, TGFβ1 is produced by a variety of cells and acts as an intercellular signaling molecule that induces the expression of cytokines and angiogenic factors, which consequently promote tumor growth, invasion and metastasis (1). Long-term reciprocal interactions between cancer cells and fibroblasts, which are a source of TGFβ1 production (34), give rise to an altered cancer cell phenotype that may affect stromal components. In the present study, the long-term exposure of the PCa cells to TGFβ1 was found to suppress THP-1 macrophage activation in a co-culture system. This result concurs with a colon cancer study in which a COX-2-degrading enzyme was upregulated by TGFβ1 (35), suggesting that the long-term exposure of PCa cells to TGFβ1 may have a similar COX-2 suppression mechanism. The current *in vitro* results may have relevance for physiological cancer tissues, wherein certain populations of cancer cells may control inflammatory cell function and gain survival advantages. A study revealed that when NF-κB signaling was repressed in TAMs, those TAMs showed cytotoxicity against tumor cells (36). In addition, normal mammary epithelial cells (MECs) exposed to TGFβ1 underwent EMT and acquired features of stromal cells. These immortalized and transformed MECs with EMT-regulated gene expression also showed increased mammosphere formation, a surrogate measure of stemness (37). Taken together, these results suggested that the long-term exposure of PCa cells to TGFβ1 may also promote a stem cell-like character. However, whether PCa with stem cell-like characteristics can suppress macrophage activity in tumors has not been fully investigated. The current *in vitro* results indicated the possibility of a macrophage inhibitory mechanism in the tumor microenvironment.

## Figures and Tables

**Table I:** qPCR analysis of human signal transduction molecules.

Pathway	Gene	Fold-change of TbL-PC3 to CoL-PC3^a^
TGFβ	CDKN1A (p21)	1.670
	CDKN1B (p27)	0.835
	CDKN2A (p16)	1.558
	CDKN2B (p15)	0.727
Phospholipase C	FOS	0.363
	ICAM1	0.959
	NOS2A	0.389
	COX2	0.257

a Fold expression changes of TbL-PC3 to CoL-PC3 cells were calculated according to the instructions of the RT^2^Profiler PCR array. COX-2, cyclooxygenase-2; TGFβ, transforming growth factor β; qPCR, quantitative polymerase chain reaction.

## Abbreviations

TGFβ1: transforming growth factor β1
COX-2: cyclooxygenase-2
EMT: epithelial mesenchymal transition
PGE_2_: prostaglandin E_2_
TAM: tumor-associated macrophage
